# The occurrence of adverse events in relation to time after registration for coronary artery bypass surgery: a population-based observational study

**DOI:** 10.1186/1749-8090-8-74

**Published:** 2013-04-11

**Authors:** Boris G Sobolev, Guy Fradet, Lisa Kuramoto, Basia Rogula

**Affiliations:** 1 The University of British Columbia, 828 West 10th Avenue, Vancouver, BC V5Z 1M9, Canada; 2 The University of British Columbia, 2251 Pandosy Street, Kelowna, BC V1Y 1T1, Canada; 3 Centre for Clinical Epidemiology and Evaluation, Vancouver Coastal Health Research Institute, 828 West 10th Avenue, Vancouver, Canada

**Keywords:** Coronary artery bypass surgery, Hospital wait list, Prioritization, Preoperative death, Emergency surgery

## Abstract

**Background:**

Our objective was to evaluate the effect of delays on adverse events while waiting for coronary artery bypass grafting (CABG).

**Methods:**

An observational study that prospectively followed patients from registration on a wait list to removal for planned surgery, death while waiting, or unplanned emergency surgery. The population-based registry provided data on 12,030 patients with a record of registration on a wait list for first-time isolated CABG surgery between 1992 and 2005.

**Results:**

In total, 104 patients died and 382 patients underwent an emergency surgery before planned CABG. The death rate was 0.5 per 1000 patient-weeks in the semiurgent group and 0.6 per 1000 patient-weeks the nonurgent group, adjusted OR = 1.07 (95% confidence interval [CI] 0.69—1.65). The emergency surgery rate of 1.2 per 1000 patient-weeks in the nonurgent group was lower compared to 2.1 per 1000 patient-weeks in the semiurgent group (adjusted OR = 0.72, 95% CI 0.54–0.97). However, the nonurgent group had a greater cumulative incidence of preoperative death than the semiurgent group for almost all weeks on the wait list, adjusted OR = 1.92 (95% CI 1.25–2.95). The surgery rate was 1.2 per 1000 patient-weeks in the nonurgent group and 2.1 per 1000 patient-weeks in the semiurgent group, adjusted OR = 0.72 (95% CI 0.54–0.97). The cumulative incidence of emergency surgery before planned CABG was similar in the semiurgent and nonurgent groups, adjusted OR = 0.88, (95% CI 0.64–1.20).

**Conclusion:**

Despite similar death rates in the semiurgent and nonurgent groups, the longer waiting times in the nonurgent group result in a greater cumulative incidence of death on the wait list compared to that in the semiurgent group. These longer waiting times also offset the lower rate of emergency surgery before planned admission in the nonurgent group so that the cumulative incidence of the emergency surgery was similar in both groups.

## Background

Delaying access to surgical procedures is a common alternative to having surplus capacity available at all times [1]. As argued elsewhere, surgical wait lists have been accepted on the ground that they provide efficient use of resources in health systems that budget the number of surgical procedures [2]. For example, cardiac services across Canada use wait lists to manage access to coronary artery bypass surgery (CABG) in periods when demand exceeds funded capacity [3-5]. Explicitly queuing patients according to urgency of required treatment is used to facilitate access to care within a clinically appropriate time. However, despite the concern that delays in necessary treatment could lead to poor clinical outcomes, the point at which the delay for CABG becomes too long has not been established [6].

Our objective was to evaluate the effect of delays on the occurrence of adverse events while waiting for CABG. In particular, we conducted an observational study to achieve a better understanding of whether longer delays for coronary artery bypass grafting contribute to worsening of the condition in less urgent patients waiting for planned CABG, and to estimate the risk of unplanned emergency surgery among these patients. We prospectively followed patients from registration on a wait list for first-time CABG to removal for planned surgery, death while waiting, or unplanned emergency surgery. We used all relevant records from the population-based registry of patients with angiographically proven coronary artery disease identified as needing bypass surgery on a non-emergency basis between 1992 and 2005. Primary comparisons have been done across synthetic cohorts of patients defined by the urgency at the decision to proceed with surgery.

## Methods

### Data sources

Data from the British Columbia Cardiac Registries (BCCR) were used to identify the study participants and their demographic, clinical and treatment characteristics. This population-based patient registry prospectively captures the date of booking request for operating room time, and the date of and reason for removal from the wait list, for all adult patients accepted for CABG in any of the four cardiac centers in the province [7]. To identify cardiac catheterization dates and coexisting medical conditions, we used each patient’s provincial health number to deterministically link BCCR records to the Canadian Institute for Health Information (CIHI) Discharge Abstract Database (DAD) [8]. To identify coexisting conditions, we used diagnoses reported in the DAD within one year prior to the booking request. Census data on the decile of median income in enumeration area were based on the postal code of the patient’s residence.

### Patients

We studied patients who had a record of registration on a wait list for first-time isolated CABG surgery from January 1, 1992 to December 31, 2005, and who had a record of catheterization procedure in the DAD. The inception cohort had 14,049 records of registration for CABG from January 1, 1991 to December 31, 2005. We excluded 567 records of patients for various reasons: procedure at registration was not isolated CABG (312), procedure at registration or at surgery was not first-time CABG (62), emergency cases at the time of registration (34), missing operating room reports (4), removed on the registration date (101), registration was on a weekend and admission was day after (14), or the patient had multiple episodes (40). We also excluded 1,452 records of patients who were registered in 1991 (797) or did not have a catheterization date (655). The remaining 12,030 records had either the surgery date or the date and reason of removal from the list without surgery.

### Primary study variable

The study variable was urgency group at registration categorized as urgent, semiurgent, and nonurgent. When placing patients on wait lists in British Columbia, Canada, all cardiac surgeons indicate the urgency of CABG according to angiographic findings, symptom severity, and left ventricular dysfunction (ejection fraction less than 50%) to ensure timing of revascularization according to the provincial guidelines: within one week for urgent procedures, within six weeks for semiurgent procedures, and within 26 weeks for nonurgent procedures [9].

### Outcomes

The primary outcomes were (1) preoperative death from all causes and (2) unplanned emergency surgery while awaiting a planned CABG. Surgeons on call made the decision to operate on patients who presented to the emergency or admitting department. All admissions from the emergency department and admissions from other locations bearing an emergency code were classified as unplanned emergency surgery. The date at which a surgeon’s office submits the operating room booking request for surgery serves as the date of registration on the list. Because scheduling is done weekly, wait-list time for each patient was computed as the number of calendar weeks from registration to removal from wait lists or end of study period. We restricted the analysis to the first 52 weeks following registration because of the lack of information to identify periods when patients were not ready for surgery, which might have contributed to extended waits.

### Potential confounders

The existing literature suggests that elderly patients are more likely to undergo revascularization as an urgent procedure [10], that smaller diameter of the coronary vessels may account for the higher risk of adverse cardiovascular events among women [11], that co-existing conditions may delay open heart surgery [12], that institutional constraints and individual care providers may affect clinical outcomes [13], that patients with a lower socioeconomic status may wait longer for cardiac surgery [14], and that changes in practice or the availability of supplementary funds may reduce the waiting time until surgery [15]. To identify comorbidities at the time of registration, we used diagnoses reported in the DAD within one year prior to registration. The reference category was defined as no coexisting conditions. The first comparison category was defined as patients with any of the following conditions at presentation: congestive heart failure, diabetes mellitus, chronic obstructive pulmonary disease, cancer, or rheumatoid arthritis [16]. The second comparison category was defined as patients presenting with other coexisting chronic conditions, as defined elsewhere [17].

Other confounders include hospital booking catheterization to address variation in standards and calendar year of surgery decision as a proxy of changes in practice and available funding. We also included the time between catheterization and surgery, the mode of admission for catheterization, urgency at admission for catheterization, which may differ substantially among hospitals affecting estimates of the total of delays in undergoing the operation [18]. The time between catheterization and registration was computed as the number of calendar weeks. The catheterization dates were obtained from the CIHI DAD and defined as the most recent diagnostic (Canadian Classification of Procedure (CCP) codes 4892–4898, 4996, 4997) or therapeutic (CCP codes 4802, 4803, 4809) catheterization performed within one year preceding and including the date of booking. We used the date of most recent catheterization procedures (diagnostic or therapeutic) because the results of this procedure are most likely linked to decision to operate [19].

### Probability of remaining on the list and weekly event rates

The probability of remaining on the list within a certain time of registration was estimated using the product-limit method [20]. Time to removal from the lists was compared across urgency groups using the log-rank test [21]. Average weekly event rates were calculated as the number of events divided by the sum of observed waiting times measured in weeks.

### Cumulative incidence of event

The cumulative incidence function (CIF) of an event is the proportion of CABG candidates experiencing the event of interest (e.g. death) instead of competing events (e.g. planned surgery) by a certain time on the wait list [22,23]. Both the event rate and the probability of remaining on the list influence the CIF. Therefore, if the CIF of an event differs between two groups when the event rates are the same, then it is the probabilities of remaining on the list that contribute to this difference. Using Gray’s test, the CIF was compared across urgency groups [24]. Further details on the cumulative incidence of event may be found in Additional file 1.

### Regression models

The effect size of urgency group on weekly rates of death and unplanned emergency surgery were estimated using discrete-time survival regression models, which naturally gives rise to the odds ratio (OR) [25]. To estimate the effect of urgency group on the cumulative incidence of death and unplanned emergency surgery, regression methods for CIF were used [26]. Further details on regression of CIF may be found in Additional file 1.

In these regression models, we adjusted for potential confounders allowing for at least 10 events per variable [27]. In the regression models for preoperative death, we adjusted for sex, age decade, comorbidities at registration, calendar period of registration, and time between catheterization and registration. In the regression models for unplanned emergency surgery, we adjusted for sex, age group, coronary anatomy at registration as a proxy for severity of coronary disease, comorbidities at registration, calendar period at registration, institution at registration, institution at catheterization, mode of admission at catheterization, urgency at admission for catheterization, and time between catheterization and registration. We performed additional analyses, in which we adjusted for socioeconomic decile in these models.

The Behavioural Research Ethics Board of the University of British Columbia approved the study protocol, Certificate of Approval H06-80651.

## Results

### Patient characteristics

Overall, this population-based study included 12,030 patients who were registered on a wait list for first-time isolated CABG surgery from January 1, 1992 to December 31, 2005. Among these patients, the majority had semiurgent status (73%), were men (83%), and between 60 to 79 years of age (68%) (Table 1). As expected, urgent patients were sicker than semiurgent or nonurgent patients because they had a higher prevalence of left main coronary artery disease (p < 0.001) and more comorbidities (p < 0.001). The institution at registration and the institution booking catheterization also differed across urgency groups (p < 0.001). Fewer nonurgent patients were admitted for catheterization through the emergency department (p < 0.001) and more tended to be elective admissions for catheterization (p < 0.001). More urgent patients were registered on a wait list within a week of catheterization (p < 0.001). Socioeconomic decile also differed across urgency groups (p = 0.04).

**Table 1:** Characteristics of 12,030 patients, registered for bypass surgery in British Columbia 1992–2005, overall and by urgency group at registration

**Characteristic**	All patients* (n = 12,030)		**Urgent (n = 739)**		**Semiurgent (n = 8,769)**		**Nonurgent (n = 2,304)**	
Sex								
Men	9981	(83.0)	599	(81.1)	7327	(83.6)	1878	(81.5)
Women	2049	(17.0)	140	(18.9)	1442	(16.4)	426	(18.5)
Age group (years)								
<50	851	(7.1)	49	(6.6)	606	(6.9)	187	(8.1)
50–59	2665	(22.2)	140	(18.9)	1946	(22.2)	541	(23.5)
60–69	4510	(37.5)	266	(36.0)	3313	(37.8)	858	(37.2)
70–79	3648	(30.3)	247	(33.4)	2652	(30.2)	657	(28.5)
≥80	356	(3.0)	37	(5.0)	252	(2.9)	61	(2.6)
Coronary anatomy at registration								
Left main	1780	(14.8)	493	(66.7)	1253	(14.3)	27	(1.2)
Multivessel†	8715	(72.4)	195	(26.4)	6673	(76.1)	1792	(77.8)
Limited‡	1535	(12.8)	51	(6.9)	843	(9.6)	485	(21.1)
Comorbidity at registration								
Major conditions§	2901	(24.1)	184	(24.9)	2084	(23.8)	556	(24.1)
Other conditions||	2856	(23.7)	217	(29.4)	2139	(24.4)	462	(20.1)
None	6273	(52.1)	338	(45.7)	4546	(51.8)	1286	(55.8)
Calendar period at registration								
1992–1996	4489	(37.3)	390	(52.8)	3239	(36.9)	822	(35.7)
1997–2001	4293	(35.7)	200	(27.1)	3049	(34.8)	1013	(44.0)
2002–2005	3248	(27.0)	149	(20.2)	2481	(28.3)	469	(20.4)
Institution at registration								
1	2668	(22.2)	137	(18.5)	1987	(22.7)	523	(22.7)
2	2873	(23.9)	258	(34.9)	2380	(27.1)	202	(8.8)
3	2914	(24.2)	62	(8.4)	1455	(16.6)	1249	(54.2)
4	3575	(29.7)	282	(38.2)	2947	(33.6)	330	(14.3)
Institution booking catheterization								
1	2759	(22.9)	152	(20.6)	2079	(23.7)	500	(21.7)
2	2775	(23.1)	249	(33.7)	2296	(26.2)	201	(8.7)
3	2037	(16.9)	36	(4.9)	1048	(12.0)	857	(37.2)
4	2798	(23.3)	211	(28.6)	2235	(25.5)	331	(14.4)
Other	1661	(13.8)	91	(12.3)	1111	(12.7)	415	(18.0)
Mode of admission for catheterization								
Emergency department	862	(7.2)	100	(13.5)	644	(7.3)	107	(4.6)
Otherwise¶	11168	(92.8)	639	(86.5)	8125	(92.7)	2197	(95.4)
Urgency at admission for catheterization								
Elective	9600	(79.8)	496	(67.1)	6920	(78.9)	2007	(87.1)
Emergency or urgent	2430	(20.2)	243	(32.9)	1849	(21.1)	297	(12.9)
Weeks between catheterization and registration								
0–1	6651	(55.3)	519	(70.2)	4743	(54.1)	1268	(55.0)
2–3	2066	(17.2)	120	(16.2)	1564	(17.8)	357	(15.5)
4–5	1041	(8.7)	40	(5.4)	811	(9.2)	174	(7.6)
6–7	642	(5.3)	17	(2.3)	483	(5.5)	131	(5.7)
≥8	1630	(13.5)	43	(5.8)	1168	(13.3)	374	(16.2)
Socioeconomic decile								
1	1160	(9.6)	77	(10.4)	818	(9.3)	246	(10.7)
2	1208	(10.0)	68	(9.2)	888	(10.1)	236	(10.2)
3	1172	(9.7)	85	(11.5)	832	(9.5)	241	(10.5)
4	1182	(9.8)	46	(6.2)	916	(10.4)	202	(8.8)
5	1122	(9.3)	82	(11.1)	825	(9.4)	194	(8.4)
6	1103	(9.2)	67	(9.1)	799	(9.1)	211	(9.2)
7	1119	(9.3)	65	(8.8)	805	(9.2)	214	(9.3)
8	1167	(9.7)	65	(8.8)	862	(9.8)	222	(9.6)
9	1138	(9.5)	75	(10.1)	819	(9.3)	233	(10.1)
10	1124	(9.3)	76	(10.3)	818	(9.3)	203	(8.8)
Unknown or missing	535	(4.4)	33	(4.5)	387	(4.4)	102	(4.4)

*Includes 218 patients for whom urgency was not provided.

†Two or three-vessel disease with stenosis of the proximal left anterior descending (PLAD) artery.

‡Two-vessel disease with no stenosis of the PLAD artery or one-vessel disease with stenosis of the PLAD artery.

§Congestive heart failure, diabetes mellitus, chronic obstructive pulmonary disease, rheumatoid arthritis, or cancer.

||Peripheral vascular disease, cerebrovascular disease, dementia, peptic ulcer disease, hemiplegia, renal disease, or liver disease.

¶Clinics or day surgery from reporting hospital, or direct patients from admitting department.

Among these patients 9,957 (82.8%) underwent planned surgery within 1 year of registration and the remaining were removed from the list for various reasons: 104 (0.9%) died, 382 (3.2%) had unplanned emergency surgery, 257 (2.1%) continued to receive medical treatment, 231 (1.9%) declined surgery, 86 (0.7%) were transferred to another surgeon or hospital, 321 (2.7%) were removed for other reasons, and 692 (5.8%) remained on the list after 52 weeks or at the end of the study period. In total, almost 500 (4%) patients had an adverse event while waiting for a planned CABG. Table 2 shows outcomes of registration for CABG across urgency groups. Among patients who underwent surgery after unplanned emergency admission, the distributions of age (p = 0.80), sex (p = 0.46), time between catheterization and registration (p = 0.29), and socioeconomic status (p = 0.28) did not seem to differ across urgency group at registration. Other characteristics differed across the groups (p < 0.001).

**Table 2:** Outcomes of registration for bypass surgery in British Columbia 1992–2005, by urgency group at registration

**Outcome**	**Urgent (n = 739)**	**Semiurgent (n = 8,769)**	**Nonurgent (n = 2,304)**	All patients (n = 12,030*)
Death before surgery, no. (%)	4 (0.5)	63 (0.7)	32 (1.4)	104 (0.9)
Unplanned emergency surgery, no. (%)	48 (6.5)	264 (3.0)	65 (2.8)	382 (3.2)
Planned surgery, no. (%)	655 (88.6)	7,512 (85.7)	1,627 (70.6)	9,957 (82.8)
Mean waiting time (STD), weeks	6 (7)	12 (10)	19 (12)	13 (11)
Median waiting time (IQR), weeks	3 (1–7)	10 (5–17)	16 (9–26)	10 (5–18)

Abbreviations: *CABG* = coronary artery bypass graft; *STD* = standard deviation; *IQR* = interquartile range.

*Includes 218 patients for whom urgency was not provided.

### Distribution of wait-list times

**Figure 1:**
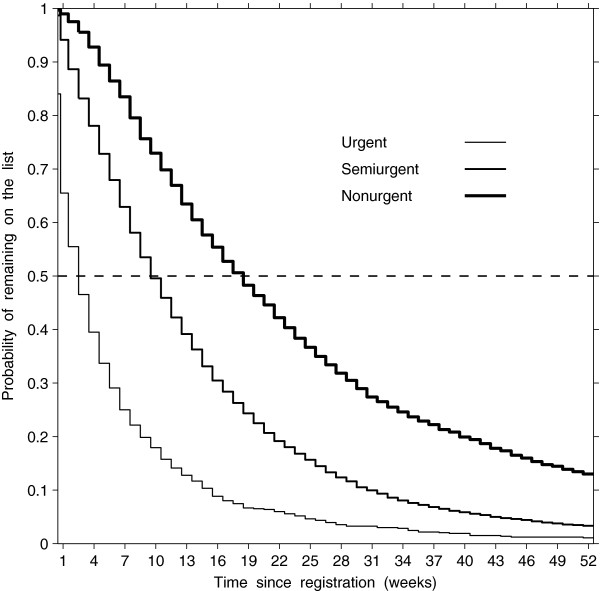
Estimated probability of remaining on wait list, by time since registration and urgency group.

**Figure 2:**
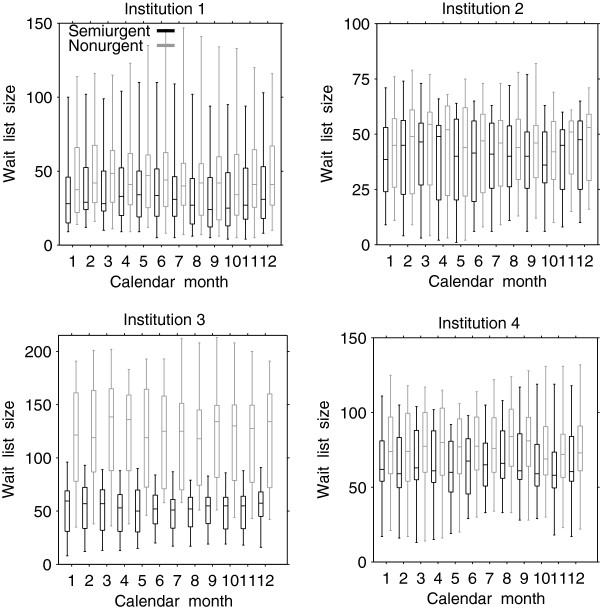
Distribution of weekly wait-list size by calendar month for semiurgent and nonurgent groups in each institution.

There were differences in the probabilities of remaining on the list by a certain week across the three urgency groups, with shorter times in higher urgency groups (log-rank test = 1329.2, df = 2, p < 0.001, Figure 1). In the nonurgent group, 75% of patients were remaining on the list after 9 weeks, 50% after 19 weeks, and 25% after 34 weeks, whereas 50% and 25% were remaining after 10 and 19 weeks in the semiurgent group and 3 and 7 weeks in the urgent group, respectively. There did not appear to be seasonality in the wait-list size for semiurgent and nonurgent groups (Figure 2). As well, there was no variation in wait-list size in the urgent group over calendar months (median wait-list size = 1; interquartile range = 0 to 2.

### Weekly preoperative event rates

In total, there were 104 deaths for 184,820 patient-weeks of remaining on the list: 4 over 4,676 patient-weeks in urgent, 63 over 123,138 patient-weeks in semiurgent, and 32 over 53,232 patient-weeks in nonurgent (Table 3). The weekly death rate varied from 0.9 per 1000 patient-weeks in the urgent group to 0.5 per 1000 patient-weeks in the semiurgent group and 0.6 per 1000 patient-weeks in the nonurgent group. After adjustment, the weekly death rate in the nonurgent group was similar to the semiurgent group (OR = 1.07, 95% confidence interval [CI] 0.69—1.65) (Table 3).

**Table 3:** Weekly rate of all-cause preoperative death, unplanned emergency surgery, and planned surgery in relation to urgency group, for patients registered for bypass surgery in 1992–2005, as measured by odds ratios derived from discrete-time survival regression models*

		**Preoperative deaths**			**Emergency surgeries**			**Planned surgeries**		
**Group**	**Total wait†**	**No. of events**	**Event rate‡ (95% CI)**	**OR§ (95% CI)**	**No. of events**	**Event rate‡ (95% CI)**	**OR|| (95% CI)**	**No. of events**	**Event rate‡ (95% CI)**	**OR|| (95% CI)**
Urgent	4,676	4	0.9 (0.0–1.7)	–	48	10.3 (7.4–13.2)	4.9 (3.4–7.2)	655	140.1 (129.3–150.8)	2.2 (2.0–2.5)
Semiurgent	123,138	63	0.5 (0.4–0.6)	1.0	264	2.1 (1.9–2.4)	1.0	7512	61.0 (59.6–62.4)	1.0
Nonurgent	53,232	32	0.6 (0.4–0.8)	1.1 (0.7–1.7)	65	1.2 (0.9–1.5)	0.7 (0.5–1.0)	1627	30.6 (29.1–32.0)	0.7 (0.6–0.7)

Abbreviations: *CI* = confidence interval, *OR* = odds ratio.

*Did not include 218 patients for whom urgency was not provided: 5 died, 5 had unplanned emergency surgery, 163 underwent planned surgery, 45 removed for other reasons.

†Waiting time measured in patient-weeks.

‡Weekly event rate was calculated as the number events divided by the sum of waiting times (per 1000 patient-weeks).

§Adjusted for sex, age decade, comorbidities at registration, calendar period of registration, and time between catheterization and registration.

||Adjusted for sex, age group, coronary anatomy, comorbidities at registration, calendar period at registration, institution at registration, institution at catheterization, mode of admission at catheterization, urgency at admission for catheterization, and time between catheterization and registration.

In total, there were 382 unplanned emergency surgeries for 184,820 patient-weeks of remaining on the list: 48 over 4,676 patient-weeks in urgent, 264 over 123,138 patient-weeks in semiurgent, and 65 over 53,232 patient-weeks in nonurgent (Table 3). The surgery rate varied from 10.3 per 1000 patient-weeks in the urgent group to 2.1 per 1000 patient-weeks in the semiurgent group and 1.2 per 1000 patient-weeks in the nonurgent group. After adjustment, the weekly surgery rate was almost five times higher in the urgent group (OR = 4.93, 95% CI 3.38–7.18) and 28% lower in the nonurgent group (OR = 0.72, 95% CI 0.54–0.97), compared to the semiurgent group (Table 3). After additional adjustment for socioeconomic decile, the effects were similar in the urgent group (OR = 4.89, 95% CI 3.33–7.16) and in the nonurgent group (OR = 0.72, 95% CI 0.53–0.98).

Table 4 shows the ORs of the all-cause preoperative death, unplanned emergency surgery, and planned surgery for the potential confounders that include patient- and center-specific factors.

**Table 4:** Odds ratios of preoperative death, unplanned emergency surgery, and planned surgery for patient and center factors, for patients registered for bypass surgery in 1992–2005, derived from discrete-time survival regression models*

**Factor**	**Preoperative deaths OR (95% CI)**	**Emergency surgeries OR (95% CI)**	**Planned surgeries OR (95% CI)**
Urgency group at registration			
Urgent	NA1	4.93 (3.38–7.18)	2.22 (2.02–2.45)
Semiurgent	1.00	1.00	1.00
Nonurgent	1.07 (0.69–1.65)	0.72 (0.54–0.97)	0.67 (0.63–0.72)
Sex			
Men	1.00	1.00	1.00
Women	0.48 (0.25–0.93)	1.06 (0.82–1.38)	0.89 (0.84–0.94)
Age decade	1.36 (1.08–1.71)	NA2	NA2
Age group (years)			
<50	NA2	1.32 (0.87–1.99)	1.10 (1.01–1.20)
50–59	NA2	1.00	1.00
60–69	NA2	1.04 (0.78–1.37)	1.05 (1.00–1.11)
70–79	NA2	1.20 (0.90–1.60)	1.07 (1.01–1.13)
≥80	NA2	0.99 (0.52–1.88)	0.84 (0.73–0.96)
Coronary anatomy at registration			
Left main	NA3	1.00	1.00
Multivessel†	NA3	1.38 (0.96–1.97)	0.83 (0.78–0.89)
Limited‡	NA3	1.93 (1.23–3.02)	0.95 (0.86–1.03)
Comorbidity at registration			
Major conditions§	1.73 (0.97–3.09)	1.01 (0.77–1.32)	0.91 (0.85–0.96)
Other conditions||	1.00	1.00	1.00
None	0.96 (0.55–1.66)	0.88 (0.67–1.16)	0.87 (0.82–0.92)
Calendar Period at registration			
1992–1996	1.33 (0.85–2.11)	1.13 (0.90–1.43)	1.16 (1.10–1.22)
1997–2001	1.00	1.00	1.00
2002–2005	0.84 (0.48–1.47)	0.74 (0.55–0.98)	1.05 (0.99–1.10)
Institution at registration			
1	NA3	1.71 (0.98–3.01)	0.78 (0.70–0.87)
2	NA3	1.00	1.00
3	NA3	0.79 (0.47–1.33)	0.54 (0.49–0.59)
4	NA3	1.23 (0.29–5.22)	1.98 (1.59–2.46)
Institution from where catheterization was booked			
1	NA3	0.70 (0.40–1.23)	1.24 (1.11–1.39)
2	NA3	1.00	1.00
3	NA3	1.00 (0.58–1.74)	0.98 (0.89–1.08)
4	NA3	0.82 (0.19–3.50)	0.73 (0.59–0.92)
Other	NA3	0.31 (0.18–0.52)	1.16 (1.07–1.25)
Mode of admission for catheterization			
Emergency department	NA3	5.66 (3.86–8.28)	0.96 (0.87–1.07)
Otherwise¶	NA3	1.00	1.00
Urgency at admission for catheterization			
Elective	NA3	1.00 (0.70–1.43)	0.85 (0.79–0.90)
Emergency or urgent	NA3	1.00	1.00
Time between catheterization and registration			
Per week	1.01 (0.98–1.03)	NA2	NA2
0–1 weeks	NA2	1.14 (0.85–1.53)	1.11 (1.04–1.17)
2–3	NA2	1.00	1.00
4–5	NA2	1.17 (0.76–1.78)	1.09 (1.00–1.18)
6–7	NA2	1.02 (0.61–1.69)	0.87 (0.79–0.97)
≥8	NA2	0.87 (0.60–1.28)	0.87 (0.81–0.94)

Abbreviations: *OR* = odds ratio, *CI* = confidence interval, *NA1* = urgent patients were excluded from this analysis, *NA2* = age was entered with alternative coding (continuous versus categorical), *NA3* = not enough events per regression variable.

*Did not include 218 patients for whom urgency was not provided: 5 died, 5 had unplanned emergency surgery, 163 underwent planned surgery, 45 removed for other reasons.

†Two or three-vessel disease with stenosis of the proximal left anterior descending (PLAD) artery.

‡Two-vessel disease with no stenosis of the PLAD artery or one-vessel disease with stenosis of the PLAD artery.

§Congestive heart failure, diabetes mellitus, chronic obstructive pulmonary disease, rheumatoid arthritis, or cancer.

||Peripheral vascular disease, cerebrovascular disease, dementia, peptic ulcer disease, hemiplegia, renal disease, or liver disease.

¶Clinics or day surgery from reporting hospital, or direct patients from admitting department.

### Cumulative incidence of event

**Figure 3:**
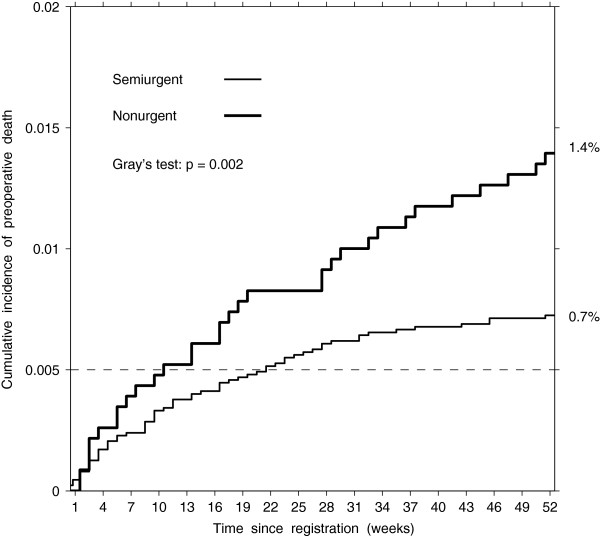
Estimated cumulative incidence of all-cause preoperative death by urgency group.

In total, 0.9% (95% CI 0.7–1.0) of patients registered for CABG died before planned surgery: 4 urgent, 63 semiurgent, 32 nonurgent, and 5 with unknown urgency (Table 5). The nonurgent group had a greater cumulative incidence of preoperative death than the semiurgent group for most weeks on the wait list (Gray’s test statistic = 9.4, df = 1, p = 0.002, Figure 3). After adjustment, the odds of death before planned surgery were 1.9 times higher in the nonurgent group compared to the semiurgent group (OR = 1.92, 95% CI 1.25–2.95) (Table 5). We attribute the higher cumulative incidence of preoperative deaths in the nonurgent group to the longer waiting times, because the death rates were similar in the semiurgent and nonurgent groups.

**Table 5:** Cumulative incidence of all-cause preoperative mortality, unplanned emergency surgery, and planned surgery in relation to urgency group, for patients registered for bypass surgery in 1992–2005, as measured by odds ratios derived from regression models for pseudovalues of cumulative incidence functions*

		**Preoperative deaths**			**Emergency surgeries**			**Planned surgeries**		
**Urgency**	**No. of patients**	**No. of events**	**% events† (95% CI)**	**OR‡ (95% CI)**	**No. of events**	**% events† (95% CI)**	**OR§ (95% CI)**	**No. of events**	**% events† (95% CI)**	**OR§ (95% CI)**
Urgent	739	4	0.5 (0.0–1.1)	–	48	6.5 (4.7–8.3)	2.5 (1.7–3.6)	655	88.6 (86.3–90.9)	3.9 (3.4–4.6)
Semiurgent	8,769	63	0.7 (0.5–0.9)	1.0	264	3.0 (2.7–3.4)	1.0	7512	85.7 (84.9–86.4)	1.0
Nonurgent	2,304	32	1.4 (0.9–1.9)	1.9 (1.3–3.0)	65	2.8 (2.1–3.5)	0.9 (0.6–1.2)	1627	70.6 (68.8–72.5)	0.5 (0.5–0.6)

Abbreviations: *CI* = confidence interval, *OR* = odds ratio.

*Did not include 218 patients for whom urgency was not provided: 5 died, 5 had unplanned emergency surgery, 163 underwent planned surgery, 45 removed for other reasons.

†At 52 weeks.

‡Odds ratio of death adjusted for sex, age decade, comorbidities at registration, calendar period of registration and time between catheterization and registration.

§Odds ratio of admission adjusted for sex, age group, coronary anatomy, comorbidities at registration, calendar period of registration, institution at registration, institution at catheterization, mode of admission at catheterization, urgency at admission for catheterization, and time between catheterization and registration.

**Figure 4:**
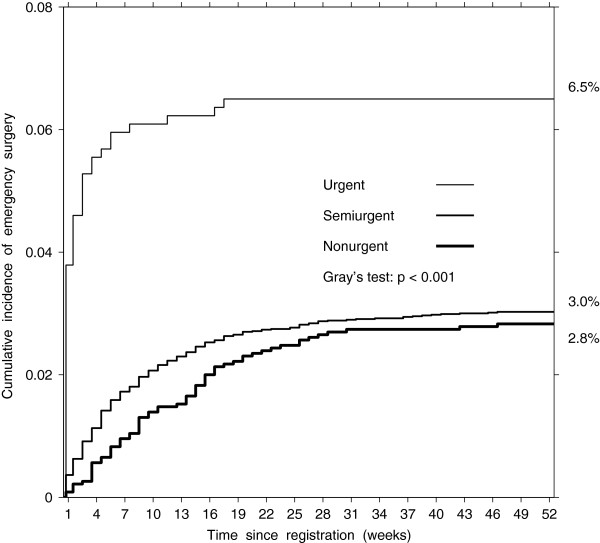
Estimated cumulative incidence of unplanned emergency surgery by urgency group.

In total, 3.2% (95% CI 2.9–3.5) of patients registered for a planned CABG had an unplanned emergency surgery: 48 urgent, 264 semiurgent, and 65 nonurgent (Table 5). The urgent group had the highest cumulative incidence of unplanned emergency surgery for all weeks on the wait list compared to the other two groups (Gray’s test statistic = 29.2, df = 2, p < 0.001, Figure 4). However, the cumulative incidences were not different between the semiurgent and nonurgent groups (Gray’s test statistic = 0.28, df = 1, p = 0.60). After adjustment, the odds of unplanned emergency surgery were 2.5 times higher in the urgent group (OR = 2.49, 95% CI 1.71–3.61) but not different in the nonurgent group (OR = 0.88, 95% CI 0.64–1.20) as compared to the semiurgent group (Table 5). After additional adjustment for socioeconomic decile, the effect did not change in the urgent group (OR = 2.45, 95% CI 1.67–3.59) and in the nonurgent group (OR = 0.87, 95% CI 0.63–1.20). The similar cumulative incidence of emergency surgery suggests that the longer waiting times in the nonurgent group offset the lower rate of emergency surgery in this group compared to the semiurgent group.

Table 6 shows the ORs of all-cause preoperative death, unplanned emergency surgery, and planned surgery for patient- and center-specific factors derived from the regression model for CIF.

**Table 6:** Odds ratios of preoperative death, unplanned emergency surgery, and planned surgery for patient and center factors, for patients registered for bypass surgery in 1992–2005, derived from regression models for cumulative incidence functions*

**Factor**	**Preoperative deaths OR (95% CI)**	**Emergency surgeries OR (95% CI)**	**Planned surgeries OR (95% CI)**
Urgency group at registration			
Urgent	NA1	2.49 (1.71–3.61)	3.94 (3.36–4.62)
Semiurgent	1.00	1.00	1.00
Nonurgent	1.92 (1.25–2.95)	0.88 (0.64–1.20)	0.52 (0.48–0.57)
Sex			
Men	1.00	1.00	1.00
Women	0.51 (0.27–0.98)	1.14 (0.87–1.49)	0.86 (0.78–0.93)
Age decade	1.26 (1.00–1.59)	NA2	NA2
Age group (years)			
<50	NA2	1.20 (0.79–1.83)	1.12 (0.98–1.28)
50–59	NA2	1.00	1.00
60–69	NA2	0.99 (0.75–1.32)	1.05 (0.97–1.15)
70–79	NA2	1.14 (0.85–1.54)	1.04 (0.95–1.13)
≥80	NA2	1.09 (0.57–2.10)	0.69 (0.56–0.85)
Coronary anatomy at registration			
Left main	NA3	0.73 (0.50–1.05)	1.36 (1.23–1.50)
Multivessel†	NA3	1.00	1.00
Limited‡	NA3	1.24 (0.89–1.73)	1.30 (1.17–1.45)
Comorbidity at registration			
Major conditions§	1.90 (1.06–3.41)	1.15 (0.87–1.51)	0.81 (0.74–0.89)
Other conditions||	1.00	1.00	1.00
None	1.08 (0.62–1.89)	1.05 (0.79–1.39)	0.77 (0.71–0.84)
Calendar Period at registration			
1992–1996	1.31 (0.83–2.07)	1.03 (0.81–1.30)	1.40 (1.30–1.51)
1997–2001	1.00	1.00	1.00
2002–2005	0.83 (0.48–1.44)	0.76 (0.57–1.03)	1.05 (0.97–1.14)
Institution at registration			
1	NA3	1.84 (1.16–2.91)	0.50 (0.42–0.58)
2	NA3	1.00	1.00
3	NA3	1.16 (0.69–1.93)	0.38 (0.33–0.44)
4	NA3	0.92 (0.33–2.62)	1.97 (1.45–2.68)
Institution from where catheterization was booked			
1	NA3	0.63 (0.40–1.01)	1.28 (1.09–1.51)
2	NA3	1.00	1.00
3	NA3	0.97 (0.57–1.66)	0.99 (0.85–1.15)
4	NA3	0.86 (0.30–2.47)	0.73 (0.53–1.00)
Other	NA3	0.26 (0.15–0.45)	1.36 (1.20–1.54)
Mode of admission for catheterization			
Emergency department	NA3	5.94 (4.05–8.71)	0.83 (0.70–0.98)
Otherwise¶	NA3	1.00	1.00
Urgency at admission for catheterization			
Elective	NA3	1.13 (0.79–1.62)	0.70 (0.63–0.77)
Emergency or urgent	NA3	1.00	1.00
Time between catheterization and registration			
Per week	1.02 (0.99–1.04)	NA2	NA2
0–1 weeks	NA2	1.07 (0.79–1.45)	1.17 (1.07–1.29)
2–3	NA2	1.00	1.00
4–5	NA2	1.14 (0.74–1.77)	1.05 (0.92–1.19)
6–7	NA2	1.08 (0.64–1.82)	0.83 (0.71–0.97)
≥8	NA2	0.96 (0.65–1.43)	0.82 (0.73–0.92)

Abbreviations: *OR* = odds ratio, *CI* = confidence interval, *NA1* = urgent patients were excluded from this analysis, *NA2* = age was entered with alternative coding (continuous versus categorical), *NA3* = not enough events per regression variable.

*Did not include 218 patients for whom urgency was not provided: 5 died, 5 had unplanned emergency surgery, 163 underwent planned surgery, 45 removed for other reasons.

†Two or three-vessel disease with stenosis of the proximal left anterior descending (PLAD) artery.

‡Two-vessel disease with no stenosis of the PLAD artery or one-vessel disease with stenosis of the PLAD artery.

§Congestive heart failure, diabetes mellitus, chronic obstructive pulmonary disease, rheumatoid arthritis, or cancer.

||Peripheral vascular disease, cerebrovascular disease, dementia, peptic ulcer disease, hemiplegia, renal disease, or liver disease.

¶Clinics or day surgery from reporting hospital, or direct patients from admitting department.

### Analysis of competing events

In total, 9,957 patients underwent planned surgery over 184,820 patient-weeks: 655 over 4,676 patient-weeks (140.1 per 1000 patient-weeks) in the urgent group, 7,512 over 123,138 patient-weeks (61.0 per 1000 patient-weeks) in the semiurgent group, and 1,627 over 53,232 patient-weeks (30.6 per 1000 patient-weeks) in the nonurgent group (Table 3). After adjustment, the weekly surgery rate was over two times higher in the urgent group (OR = 2.22, 95% CI 2.02–2.45) and 33% lower in the nonurgent group (OR = 0.67, 95% CI 0.63–0.72), compared to the semiurgent group. After additional adjustment for socioeconomic decile, the effects were similar in the urgent group (OR = 2.24, 95% CI 2.03–2.48) and in the nonurgent group (OR = 0.68, 95% CI 0.64–0.72).

**Figure 5:**
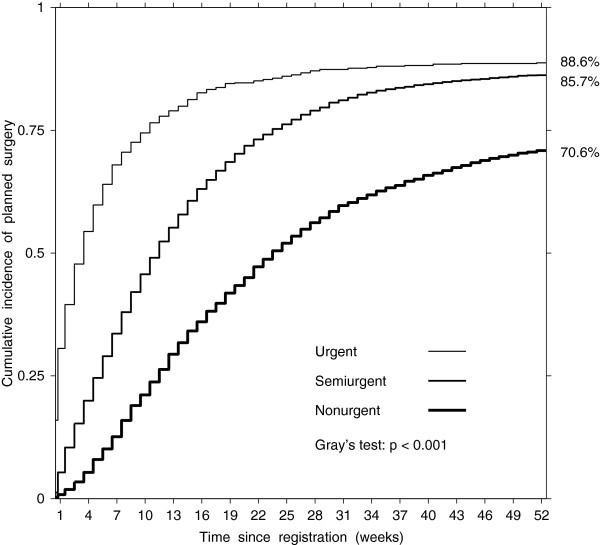
Estimated cumulative incidence of planned surgery by urgency group.

Overall, 82.8% (95% CI 82.1–83.4) of patients registered for CABG underwent planned surgery: 88.6% (95% CI 86.3–90.9) in the urgent group, 85.7% (95% CI 84.9%–86.4%) in the semiurgent group, and 70.6% (95% CI 68.8–72.5) in the nonurgent group (Table 5). The urgent group had the highest cumulative incidence of planned surgery for all weeks on the wait list, followed by the semiurgent group and the nonurgent group had the lowest cumulative incidence (Gray’s test statistic = 539.6, df = 2, p < 0.001, Figure 5). After adjustment, the odds of planned surgery were about four times higher in the urgent group (OR = 3.94, 95% CI 3.36–4.62) and 48% lower in the nonurgent group (OR = 0.52, 95% CI 0.48–0.57) as compared to the semiurgent group (Table 5). After additional adjustment for socioeconomic decile, the effect did not change in the urgent group (OR = 3.95, 95% CI 3.35–4.64) and in the nonurgent group (OR = 0.52, 95% CI 0.48–0.57).

## Discussion

Our results confirm that queuing patients according to urgency of treatment contributes to a higher proportion of preoperative death among CABG candidates in the less urgent category. Even though the death rate was similar in the nonurgent and semiurgent groups, 0.5 versus 0.6 per 1000 patient-weeks, patients in the non-urgent group were remaining on the list longer, which resulted in a doubled cumulative incidence of all-cause death compared to the semiurgent group (OR = 1.92, 95% CI 1.25–2.95). Our results also suggest that longer waiting times offset the lower rate of emergency surgery before planned admission in the nonurgent group than in the semiurgent group, 1.2 versus 2.1 per 1000 patient-weeks, so that the cumulative incidence of the emergency surgery is similar (OR = 0.9, 95% CI 0.6–1.2).

We studied patients who were registered on a wait list for first-time isolated CABG surgery. Patients who underwent the procedure by direct admission to hospital on a non-emergency basis were not included in the analysis. Considering that cardiac surgeons in British Columbia have discretion for direct admission of their patients, these two groups may be incomparable in terms of their clinical presentation and waiting time [28]. Our analysis focused on preoperative events, such as death on wait list. Therefore, we did not report on postoperative events.

Our study had several limitations. First, because of its observational nature, patient and clinical factors that influence the risk of preoperative events might have different distributions across urgency groups. To address this issue, we used regression adjustment for measured factors. We also attempted to capture unmeasured factors by using calendar period as a proxy for changes in the population of CABG patients. Even so, these techniques may not fully address the issue of potential confounding due to unmeasured factors. The time to surgery may differ between patients treated by surgeons with high volume of CABG procedures and surgeons who perform a diverse range of cardiac procedures. Second, a potential concern is misclassification of the recorded urgency for treatment, because surgeons may select patients from the wait lists based on various considerations, such as best use of operating time or the availability of hospital resources. Therefore, the occurrence of preoperative events might have been influenced by the individual surgeon’s threshold for accepting a patient for non-urgent treatment. Third, the time to surgery may reflect patient and clinician decisions following the registration for surgery, in addition to system and clinical factors. We did not have this information. Therefore, our results can be interpreted only as the net effect of timing of surgery in a possibly self-selected patient population. Fourth, care received outside of the Canadian health care system (e.g., paid for separately) may impact waiting times and outcomes. In our study, there was no mechanism to ascertain such cases, if any, separately from the registry data. Finally, several studies have shown that coexisting conditions are underreported in administrative databases for patients discharged after cardiovascular procedures [29-31]. As such, the effect of timing of surgery may be attributable to unmeasured clinical factors, which might result in an upward bias in survival effect for those unfit for the operation.

## Conclusion

**Figure 6:**
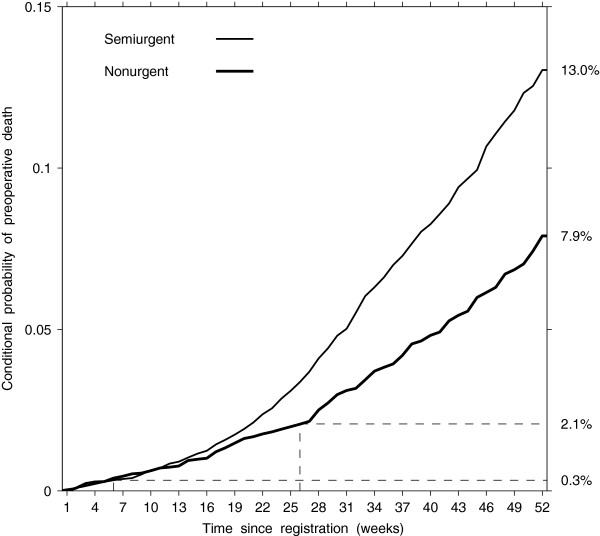
Estimated conditional probability of preoperative death by urgency group.

In conclusion, the contribution of this article is two-fold. First, we present the perspective of health service research on studying the risk of adverse events while waiting for recommended treatment. The estimates of cumulative incidence of adverse events on CABG wait lists, which is a function of both the event rate and the probability of remaining on the list, may be useful to hospital managers. Our results provide evidence for capacity planning in managing access to CABG that would minimize the preoperative adverse events associated with treatment delay, if unavoidable. For example, the point at which the wait for CABG becomes too long can be established as the period by the end of which, for a given surgical capacity, the proportion of preoperative deaths exceeds a safety standard accepted in the health system, e.g. postoperative in-hospital mortality in this patient population. Second, we provide data on risks associated with the anticipated delays in undergoing the recommended coronary revascularization. In deciding on the duration of time that non-emergency treatment can be delayed safely, policy-makers may find it useful to measure the risk of preoperative death among those who remain untreated by a certain time after registration on a wait list. For example, Figure 6 shows that, conditional on not having undergone CABG by the time recommended by the provincial guidelines, the risk of preoperative death reaches 0.3% for the semiurgent group and 2.1% for the nonurgent group.

## Competing interests

The authors declare that they have no competing interests.

## Authors’ contributions

BS conceived the study concept and design, participated in analysis and interpretation, and drafted the manuscript. GF participated in data acquisition and critically revised the manuscript. LK participated in analysis and interpretation, and drafted the manuscript. BR performed statistical analysis and drafted the manuscript. All authors read and approved the final manuscript.

## Supplementary Material

Additional file 1 Methods for cumulative incidence of event. Click here for file

## References

[B1] Thomas SJ Williams MV Burnet NG Baker CR How much surplus capacity is required to maintain low waiting times? Clin Oncol (R Coll Radiol) 2001 13 24 28 11292132 10.1053/clon.2001.9210

[B2] Fierlbeck K Health care in Canada: A Citizen’s guide to policy and politics 2011 Toronto: University of Toronto Press

[B3] Morgan CD Sykora K Naylor CD Analysis of deaths while waiting for cardiac surgery among 29,293 consecutive patients in Ontario, Canada Heart 1998 79 345 349 9616340 PMC1728656

[B4] Ray AA Buth KJ Sullivan JA Johnstone DE Hirsch GM Waiting for cardiac surgery: results of a risk-stratified queuing process Circulation 2001 104 I92 I98 10.1161/hc4301.098011 11568037

[B5] Sampalis J Boukas S Liberman M Reid T Dupuis G Impact of waiting time on the quality of life of patients awaiting coronary artery bypass grafting CMAJ 2001 165 429 433 11531051 PMC81367

[B6] Sobolev BG Fradet G Delays for coronary artery bypass surgery: how long is too long? Expert Rev Pharmacoecon Outcomes Res 2008 8 27 32 10.1586/14737167.8.1.27 20528353

[B7] Sobolev B Levy A Hayden R Kuramoto L Does wait-list size at registration influence time to surgery? analysis of a population-based cardiac surgery registry Health Serv Res 2006 41 23 39 10.1111/j.1475-6773.2005.00459.x 16430599 PMC1681524

[B8] Chamberlayne R Green B Barer ML Hertzman C Lawrence WJ Sheps SB Creating a population-based linked health database: a new resource for health services research Can J Public Health 1998 89 270 273 9735524 10.1007/BF03403934 PMC6990342

[B9] Canadian Institute for Health Information Wait times in Canada - A summary, 2012 2012 Ottawa, Canada: Canadian Institute for Health Information https://secure.cihi.ca/free_products/WaitTimesSummary2012_EN.pdf

[B10] Christenson JT Simonet F Schmuziger M The influence of age on the outcome of primary coronary artery bypass grafting J Cardiovasc Surg (Torino) 1999 40 333 338 10412916

[B11] O’Connor NJ Morton JR Birkmeyer JD Olmstead EM O’Connor GT Effect of coronary artery diameter in patients undergoing coronary bypass surgery. Northern New England cardiovascular disease study group Circulation 1996 93 652 655 10.1161/01.CIR.93.4.652 8640991

[B12] Naylor CD Baigrie RS Goldman BS Basinski A Assessment of priority for coronary revascularisation procedures Lancet 1990 335 1070 1073 10.1016/0140-6736(90)92640-4 1970377

[B13] DeLong ER Nelson CL Wong JB Pryor DB Peterson ED Lee KL Mark DB Califf RM Pauker SG Using observational data to estimate prognosis: an example using a coronary artery disease registry Stat Med 2001 20 2505 2532 10.1002/sim.930 11512139

[B14] Pell JP Pell AC Norrie J Ford I Cobbe SM Effect of socioeconomic deprivation on waiting time for cardiac surgery: retrospective cohort study BMJ 2000 320 15 18 10.1136/bmj.320.7226.15 10617517 PMC27247

[B15] Levy A Sobolev B Hayden R Kiely M FitzGerald M Schechter M Time on wait lists for coronary bypass surgery in British Columbia, Canada, 1991–2000 BMC Health Serv Res 2005 5 22 10.1186/1472-6963-5-22 15766381 PMC1079832

[B16] Naylor CD Levinton CM Baigrie RS Adapting to waiting lists for coronary revascularization. Do canadian specialists agree on which patients come first? Chest 1992 101 715 722 10.1378/chest.101.3.715 1541137

[B17] Romano PS Roos LL Jollis JG Adapting a clinical comorbidity index for use with ICD-9-CM administrative data: differing perspectives J Clin Epidemiol 1993 46 1075 1079 10.1016/0895-4356(93)90103-8 8410092

[B18] Legare JF Li D Buth KJ How established wait time benchmarks significantly underestimate total wait times for cardiac surgery Can J Cardiol 2010 26 e17 e21 10.1016/S0828-282X(10)70337-8 20101361 PMC2827229

[B19] King KM Ghali WA Faris PD Curtis MJ Galbraith PD Graham MM Knudtson ML Sex differences in outcomes after cardiac catheterization - effect modification by treatment strategy and time JAMA 2004 291 1220 1225 10.1001/jama.291.10.1220 15010443

[B20] Bland JM Altman DG Survival probabilities (the kaplan-meier method) BMJ 1998 317 1572 10.1136/bmj.317.7172.1572 9836663 PMC1114388

[B21] Bland JM Altman DG The logrank test BMJ 2004 328 1073 10.1136/bmj.328.7447.1073 15117797 PMC403858

[B22] Sobolev BG Kuramoto L Analysis of waiting-time data in health services research 2007 New York: Springer

[B23] Sobolev B Kuramoto L Levy A Hayden R Sobolev B Kuramoto L Levy A Hayden R Methods for studying adverse events on surgical wait lists Health Serv Outcome Res Meth 2006 6 139 151 10.1007/s10742-006-0010-3

[B24] Gray RJ A class of K-sample tests for comparing the cumulative incidence of a competing risk Ann Stat 1988 16 1141 1154 10.1214/aos/1176350951

[B25] Cox DR Oakes D Analysis of survival data 1984 London: Chapman Hall

[B26] Klein JP Andersen PK Regression modeling of competing risks data based on pseudovalues of the cumulative incidence function Biometrics 2005 61 223 229 10.1111/j.0006-341X.2005.031209.x 15737097

[B27] Peduzzi P Concato J Kemper E Holford TR Feinstein AR A simulation study of the number of events per variable in logistic regression analysis J Clin Epidemiol 1996 49 1373 1379 10.1016/S0895-4356(96)00236-3 8970487

[B28] Sobolev BG Fradet G Kuramoto L Sobolyeva R Rogula B Levy AR Evaluation of supply-side initiatives to improve access to coronary bypass surgery BMC Health Serv Res 2012 12 311 10.1186/1472-6963-12-311 22963283 PMC3515401

[B29] Humphries KH Rankin JM Carere RG Buller CE Kiely FM Spinelli JJ Co-morbidity data in outcomes research: are clinical data derived from administrative databases a reliable alternative to chart review? J Clin Epidemiol 2000 53 343 349 10.1016/S0895-4356(99)00188-2 10785564

[B30] Austin PC Daly PA Tu JV A multicenter study of the coding accuracy of hospital discharge administrative data for patients admitted to cardiac care units in ontario Am Heart J 2002 144 290 296 10.1067/mhj.2002.123839 12177647

[B31] Quan H Parsons GA Ghali WA Validity of information on comorbidity derived rom ICD-9-CCM administrative data Med Care 2002 40 675 685 10.1097/00005650-200208000-00007 12187181

