# Supplementary material for: The occurrence of adverse events in relation to time after registration for coronary artery bypass surgery: a population-based observational study
Source: J Cardiothorac Surg. 2013 Apr 11;8:74. doi: 10.1186/1749-8090-8-74 (PMC3639061; doi:10.1186/1749-8090-8-74)
Supplement: Additional file 1 — Methods for cumulative incidence of event. [file 1749-8090-8-74-S1.doc]

# Appendix A – Methods for cumulative incidence of event

## Cumulative incidence of event

The time-dependent, cumulative incidence of an event was used to characterize the marginal probability that an event while awaiting surgery occurs on or before a certain wait-list week in the presence of competing wait-list events [1]. We interpret the cumulative incidence of preoperative death as the proportion of CABG candidates dying before planned surgery, which accrues over wait-list time when the patients can be removed from the list due to planned surgery, death, unplanned emergency surgery, or other competing events [2]. Similarly, we interpret the cumulative incidence of unplanned emergency surgery as the proportion of unplanned emergency surgery before planned surgery. The cumulative incidence function (CIF) of an event is defined as the integration over time of the product of the weekly event rate and the probability of remaining on the list [3]. Therefore, if the CIF of an event differs between two groups while the event rates are the same, then it is the probabilities of remaining on the list that contribute to this difference. The CIF of an event and its standard errors were estimated using nonparametric methods [4]. Using Gray’s test, the CIF was compared across urgency groups [5].

## Regression models

The effect size of urgency group on weekly rates of death and unplanned emergency surgery were estimated using discrete-time survival regression models, which naturally gives rise to the odds ratio (OR) [6]. We used the discrete-time survival analysis because time on a wait list is inherently discrete and best measured as the number of weekly scheduling cycles [7]. We represented time to event by a sequence of binary variables that indicated if the patient experienced the event of interest on the list at a certain week. The likelihood function of such indicators can be factored into the conditional probabilities of event during a certain week among those remaining on the list [8]. To fit a pool of binary regression models with the logit link developed for each patient by using the maximum likelihood method, it was assumed that binary indicators were independent across patients.

To estimate the effect of urgency group on the cumulative incidence of death and unplanned emergency surgery, regression methods for pseudovalues of CIF were used [9]. Pseudovalues of CIF of event are computed in the presence of planned surgery and other competing events at all distinct, observed event times. For each patient, the CIF pseudovalues correspond to a series of binary variables equal to 0 before and 1 at or after the event of interest in the absence of censoring. Using the generalized estimation equations, the CIF was modeled with adjustment for subject-level correlation between pseudovalues. The working weight matrix was fixed and estimated as a product-moment correlation matrix among pseudovalues of the CIF.

In these regression models, we adjusted for potential confounders allowing for at least 10 events per variable [10]. In the regression models for preoperative death, we adjusted for sex, age decade, comorbidities at registration, calendar period of registration, and time between catheterization and registration. In the regression models for unplanned emergency surgery, we adjusted for sex, age group, coronary anatomy at registration as a proxy for severity of coronary disease, comorbidities at registration, calendar period at registration, institution at registration, institution at catheterization, mode of admission at catheterization, urgency at admission for catheterization, and time between catheterization and registration. We performed additional analyses, in which we adjusted for socioeconomic decile in these models.

Reference List

1. Sobolev BG, Kuramoto L: *Analysis of Waiting-Time Data in Health Services Research.* New York: Springer; 2007.

2. Sobolev B, Kuramoto L, Levy A, Hayden R, Sobolev B, Kuramoto L, Levy A, Hayden R: **Methods for studying adverse events on surgical wait lists.** *Health Services and Outcomes Research Methodology* 2006, **6:**139-151.

3. Bryant J, Dignam JJ: **Semiparametric models for cumulative incidence functions.** *Biometrics* 2004, **60:**182-190.

4. Pepe MS, Mori M: **Kaplan-Meier, marginal or conditional-probability curves in summarizing competing risks failure time data.** *Stat Med* 1993, **12:**737-751.

5. Gray RJ: **A class of K-sample tests for comparing the cumulative incidence of a competing risk.** *The Annals of Statistics* 1988, **16:**1141-1154.

6. Cox DR, Oakes D: *Analysis of survival data.* London: Chapman Hall; 1984.

7. Sobolev B, Brown P, Zelt D, Kuramoto L: **Waiting time in relation to wait-list size at registration: statistical analysis of a waiting-list registry.** *Clin Invest Med* 2004, **27:**298-305.

8. Allison PD: **Discrete-time methods for the analysis of event histories.** *Sociological Methodology* 1982,61-98.

9. Klein JP, Andersen PK: **Regression modeling of competing risks data based on pseudovalues of the cumulative incidence function.** *Biometrics* 2005, **61:**223-229.

10. Peduzzi P, Concato J, Kemper E, Holford TR, Feinstein AR: **A simulation study of the number of events per variable in logistic regression analysis.** *Journal of Clinical Epidemiology* 1996, **49:**1373-1379.
